# Enhancing Mathematics Learning for Students with Intellectual and Developmental Disabilities in China: A Qualitative Study of Instructional Support

**DOI:** 10.3390/jintelligence14020018

**Published:** 2026-01-28

**Authors:** Tingrui Yan, Yaoqiong Jin

**Affiliations:** Special Education Department, Faculty of Education, East China Normal University, Zhongshan North Street, No.3663, Shanghai 200062, China; tryan@ed.ecnu.edu.cn

**Keywords:** mathematics teaching and learning, intellectual and developmental disabilities (IDD), special education, instructional support, China

## Abstract

This study explored how mathematics teachers in Chinese special schools provide instructional support to primary-aged students with intellectual and developmental disabilities (IDD). The types, characteristics, and classroom implementation processes of such support were identified to address a gap in the literature regarding subject-specific instructional practices in special education settings. A qualitative research design using interpretative phenomenological analysis (IPA) was employed. Five mathematics teachers from special schools in Shanghai participated in the study. Data were collected through 15 video-recorded classroom observations and five semi-structured interviews. Thematic analysis was conducted to identify key patterns of instructional support. The analysis revealed five core domains of instructional support for students with IDD: (1) comprehension facilitation through simplified explanations, real-life connections, and visual scaffolding; (2) responding to tasks involving prompts, modeling, and hand-over-hand support; (3) maintaining attention using individual and collective cues; (4) sustaining motivation through praise, encouragement, and second-chance opportunities; and (5) regulating behavior such as verbal restraint, physical proximity, and attention redirection. The findings contribute to a deeper understanding of effective instructional support tailored to students with IDD.

## 1. Introduction

Mathematics, as a fundamental curriculum area for students with Intellectual and Developmental Disabilities (IDD), plays a pivotal role in enhancing their daily life skills and facilitating successful social integration. Students with IDD often face considerable challenges in mathematics learning, including difficulties in basic arithmetic operations, comprehending abstract mathematical concepts, and applying learned skills across different contexts (Cheong et al., 2017; Geary, 2004; Z. Zhang et al., 2023). These challenges frequently result in significant impediments to academic progression and negatively affect their overall quality of life (San Martin et al., 2024). Given these realities, providing effective mathematics instruction tailored to the specific needs of students with IDD is critically important.

Instructional support, characterized by targeted teaching strategies and individualized assistance, is central to addressing these learning barriers (Lerang et al., 2025). Previous research has consistently shown that higher-quality instructional support is associated with improved academic outcomes, enhanced cognitive functioning, and better social-emotional development among students (Pianta et al., 2008; Allen et al., 2013; Lerang et al., 2024). However, this body of research has largely examined instructional support at a general pedagogical level or within inclusive classroom settings, with comparatively limited attention to how instructional support is enacted in subject-specific domains such as mathematics, particularly in special school contexts. As a result, there remains insufficient understanding of how mathematics teachers in special education settings design, interpret, and implement instructional support to address the distinctive cognitive and behavioral challenges associated with mathematics learning for students with IDD. In the Chinese context, nearly half of all students with IDD, especially those with severe and profound disabilities, are educated in special schools (Yan & Peng, 2026). These schools serve a distinct population with complex support needs, and their instructional practices are shaped by institutional structures, resource constraints, and the cognitive profiles of the students they serve (Fu et al., 2022; Yan et al., 2024). Yet empirical research specifically examining instructional support for mathematics learning within Chinese special schools remains scarce, leaving a critical gap in both mathematics education and special education literature.

To address this research gap, the present study focuses on instructional support for students with IDD in mathematics classrooms within Chinese special schools. By adopting an in-depth case study approach, this study aims to provide empirically grounded insights into the nature, structure, and contextual characteristics of instructional support in special education settings, thereby contributing to a more nuanced and contextually relevant understanding of instructional practices in the field of special education research.

### 1.1. Barriers of Mathematics Learning for Students with IDD

Students with IDD frequently encounter substantial challenges in learning mathematics. Compared to their typically developing peers, they often struggle with acquiring basic numerical skills, understanding mathematical concepts, performing arithmetic operations, and applying learned knowledge to real-life contexts (Polo-Blanco et al., 2024; Rousselle & Noël, 2007). For example, Bullen et al. (2020) found that children with autism spectrum disorder (ASD) and those with ADHD exhibited significant and comparable delays in both problem-solving and calculation abilities, and that lower verbal IQ was associated with lower math achievement across all groups. These persistent difficulties not only hinder their academic progress but also affect their confidence, motivation, and participation in classroom activities. As mathematics serves as a foundation for daily problem-solving and independent living, such barriers can have long-term implications for their social inclusion and quality of life.

A major source of these challenges lies in the cognitive characteristics commonly associated with IDD. Students with IDD often have limitations in abstract reasoning, symbolic thinking, and flexible problem-solving (Halder et al., 2023). Restricted working memory makes it difficult to retain and manipulate numerical information, especially in multi-step calculations (Van der Molen et al., 2014). Moreover, difficulties in communication, social interaction, and emotional self-regulation add further complexity to their learning process, particularly in collaborative or verbally mediated mathematical tasks (Saini et al., 2024).

Nevertheless, these cognitive barriers do not fully determine learning outcomes. Increasing research shows that the quality and appropriateness of instructional support can play a decisive role in shaping mathematics learning trajectories for students with IDD (Brave et al., 2025; Snodgrass et al., 2016). In classrooms with insufficient instructional support, generic teaching methods and the lack of individualized instruction often amplify students’ cognitive difficulties. For instance, whole-class instruction and rigidly tiered strategies may overlook the diverse needs of students with moderate to severe disabilities, resulting in disengagement and academic frustration. In contrast, when students receive responsive, well-adapted instructional support, including scaffolding, multi-sensory materials, and differentiated tasks, many can overcome specific learning barriers and make meaningful progress (Sutherland et al., 2023).

Given the interplay between internal cognitive limitations and external instructional factors, it is crucial to examine how instructional support is implemented to meet the unique needs of students with IDD. Yet, current research lacks a detailed understanding of how instructional support functions in real-world special education classrooms, especially in subject-specific domains like mathematics. Therefore, the investigation into instructional support is essential for advancing theoretical understanding of teaching and learning processes in special education contexts, particularly as they relate to mathematics instruction for students with IDD.

### 1.2. Instructional Support for Mathematics Learning for Students with IDD

Instructional support is defined as a constellation of teacher practices designed to enhance students’ cognitive development and learning outcomes through structured academic interactions (Allen et al., 2013; Pianta et al., 2012). It encompasses various strategies such as providing process-oriented feedback, promoting higher-order thinking, using engaging and differentiated instructional materials, and presenting new content within meaningful, real-world contexts (Hafen et al., 2015). These practices are particularly important for students with IDD, who require explicit, repeated, and scaffolded instruction to build foundational mathematical skills.

Within the context of mathematics learning, instructional support is not only about delivering content but also about how teachers mediate students’ understanding. Effective mathematics instruction for students with IDD often involves the use of multi-sensory methods, visual aids, and step-by-step scaffolding to help students conceptualize abstract ideas. Research highlights that when teachers model problem-solving processes, ask open-ended questions, and provide immediate, specific feedback, students are more likely to persist in tasks and develop a deeper understanding of mathematical concepts (Pettigrew et al., 2013). Moreover, studies have consistently demonstrated the positive impact of instructional support on student outcomes. For example, high-quality instructional interactions have been linked to improved academic achievement, enhanced engagement, and reduced off-task behaviors (Ertesvåg et al., 2022; Lerang et al., 2024; Perlman et al., 2016). Particularly for students with IDD, whose learning trajectories are highly sensitive to the quality of teacher interaction, targeted feedback and thoughtfully structured learning experiences can significantly mitigate the effects of cognitive impairments and promote meaningful mathematical progress.

A growing body of research has investigated instructional strategies designed to support mathematics learning among students with IDD, with a predominant focus on experimental and quasi-experimental intervention studies. These studies have identified a range of effective teaching methods that can enhance students’ mathematical understanding and problem-solving performance. For example, the Virtual–Representational–Abstract (VRA) framework has been widely adopted in intervention research and successfully integrated into classroom instruction. Findings show that VRA-based instruction improves students’ accuracy in solving mathematical problems and supports the retention of skills even after visual aids are withdrawn (E. C. Bouck et al., 2024; Root et al., 2021). Similarly, Schema-Based Instruction (SBI) has demonstrated superior effectiveness over general strategy instruction in enhancing word problem-solving performance among students at risk for math failure (Cook et al., 2020). In the domain of metacognitive support, presenting worked examples combined with explicit cognitive strategy instruction has been shown to result in higher problem-solving accuracy and improved learning outcomes compared to conventional instruction, both immediately and over time (Chung & Tam, 2005). Additionally, the SOLVE strategy, derived from heuristic-based approaches, has been found to significantly improve the problem-solving strategies and computation scores of students with learning disabilities (Freeman-Green et al., 2015).

Despite these promising findings, many of these studies tend to rely on structured, decontextualized intervention designs, often conducted in highly controlled environments. They typically emphasize measurable outcomes while overlooking the contextual, relational, and cultural dimensions of teaching practices. For instance, low instructional support scores in special education classrooms are sometimes interpreted as indicators of poor teaching quality. Yet such scores may fail to account for the nuanced needs of students—for example, the intentional use of frequent breaks for students with emotional or behavioral disorders, which, though reducing instructional time, may be developmentally appropriate and pedagogically sound (Blatchford et al., 2009; Luo, 2020). Consequently, there is a critical need for qualitative, context-sensitive research that explores how instructional support unfolds in real classroom settings. Such approaches can complement quantitative findings by revealing how teachers make moment-to-moment decisions, adapt strategies in response to student needs, and negotiate institutional and cultural constraints—insights that are essential for developing more realistic, sustainable models of instructional support in mathematics education for students with IDD.

### 1.3. Mathematics Instructional Support for IDD Students in Special School in China

Although international research has extensively examined instructional support for students with IDD, most studies have focused primarily on inclusive educational settings (Carabajal et al., 2017; Mumford & Chandler, 2009). Instructional practices and support dynamics in Chinese special schools—fully segregated educational contexts—are notably different from those in inclusive environments, implying potentially distinct impacts on the effectiveness of support provided.

Within the context of special education in China, a considerable number of students with moderate to severe IDD attend specialized schools exclusively catering to their educational needs (Alduais & Deng, 2019). The mathematics curriculum framework in Chinese special schools significantly differs from mainstream and international settings. According to China’s Compulsory Education Mathematics Curriculum Standards for Special Schools (2016 edition), abstract mathematical content, such as algebraic operations, is substantially reduced or replaced by contextually meaningful modules (e.g., supermarket shopping, daily budgeting), thereby forming a unique alternative curriculum specifically adapted to students’ practical living skills (Xin et al., 2024; Feng, 2022). Furthermore, teaching in Chinese classrooms typically follows a teacher-led collective instructional model, characterized by structured lessons and a disciplined learning atmosphere (L. Zhang et al., 2020). While this approach maintains classroom order and student focus, it often results in less systematic attention to individual students’ conceptual contributions or adaptive instructional adjustments (Lim, 2007; Zhou et al., 2023). Additionally, pedagogical practices in Chinese mathematics classrooms tend to emphasize symbolic representation and structured problem-solving routines, contrasting with Western practices that frequently utilize verbal explanations, graphical representations, and open-ended inquiry (Cai & Lester, 2007).

Importantly, current empirical evidence on the instructional support within Chinese special schools is inconsistent. Some studies report relatively effective teaching practices and appropriate instructional adaptations, whereas others indicate that students with IDD commonly receive insufficient or misaligned instructional support (S. Huang et al., 2023; Özdemir & Kılıç, 2023). These divergent findings may reflect the dynamic and context-dependent nature of instructional support in Chinese special education schools, shaped by variations in teacher expertise, institutional resources, regional policies, and student characteristics. Therefore, there remains a critical need for in-depth qualitative research to explore the realities of instructional support within the unique context of Chinese special schools.

### 1.4. The Present Study

Given the identified gaps in the literature and the unique context of Chinese special education, the primary purpose of this study is to systematically explore how mathematics teachers provide instructional support for students with IDD in special schools. While existing research has demonstrated the effectiveness of various instructional strategies, far less is known about how instructional support is enacted as a dynamic and situated process in teachers’ everyday classroom practice.

In this study, instructional support is conceptualized not as a static set of teaching techniques, but as a constellation of context-sensitive actions that teachers employ during real-time classroom interactions. To address the limited understanding of teachers’ lived experiences of providing instructional support in mathematics classrooms, this study adopts a qualitative approach to capture how instructional support is experienced, interpreted, and enacted by teachers in authentic instructional contexts. By focusing on mathematics teaching in Chinese special schools, the study aims to illuminate the types, characteristics, and practical implementation processes of instructional support as they unfold in everyday classroom interactions, thereby offering a process-oriented and contextually grounded account of instructional support in special education.

Accordingly, the following research questions guided this investigation:

(1) What types of instructional support do mathematics teachers provide to students with IDD in special schools?
(2) How do teachers enact and adapt instructional support during real-time classroom interactions to address the cognitive and behavioral challenges of students with IDD?

## 2. Materials and Methods

### 2.1. Methodological Approach

A qualitative research design was deemed most suitable for this study given its exploratory aim to deeply understand the dynamic processes involved in instructional support within mathematics education contexts. Specifically, interpretative phenomenological analysis (IPA) was selected, not only as an analytic technique, but as an epistemological approach grounded in phenomenology and interpretivism, due to its strong emphasis on examining how individuals actively make sense of lived experiences and the meaning-making processes embedded within these experiences (Murray & Wilde, 2020). Given that the present study seeks to understand how teachers interpret, experience, and enact instructional support in their everyday classroom practice, IPA is particularly appropriate for addressing the research questions of this study. Although IPA has been primarily developed within Western qualitative research traditions, it has increasingly been applied in non-Western educational contexts, including Chinese education, demonstrating its cross-cultural applicability and epistemological flexibility (Cutri, 2025).

Employing semi-structured interviews combined with video-recorded classroom observations enabled the collection of rich, multidimensional data. These methods facilitated the exploration of teachers’ lived experiences, instructional practices, belief systems as well as how these experiences are situated within specific classroom interactions and institutional contexts. Rather than treating instructional support as a predefined set of strategies, this study foregrounds teachers’ subjective interpretations and professional judgments as they respond to students’ cognitive and behavioral needs in real time. Through participants’ detailed narratives, IPA allows researchers to uncover nuanced insights into complex, under-explored educational phenomena (Goddard & Cook, 2021).

In the current study, the phenomenon of interest was the lived experience of mathematics teachers providing instructional support for students with IDD in special schools. IPA was applied to interpret teachers’ accounts through an idiographic and interpretative analytic process, engaging participants in the co-construction of meaning. Consistent with IPA’s idiographic commitment, each participant’s interview and corresponding classroom observations were first analyzed in depth on a case-by-case basis, followed by the identification of patterns of convergence and divergence across cases through an iterative and reflexive process. This analytic approach reflects the double hermeneutic central to IPA, whereby researchers seek to make sense of participants’ own sense-making of their instructional experiences (Barrett-Rodger et al., 2023), and enabled a focused examination of how teachers perceived and responded to students’ cognitive, emotional, and behavioral states during instructional support in authentic classroom settings.

### 2.2. Participants

Mathematics teachers from special schools for children with IDD in Shanghai were selected using purposive sampling. This sampling strategy was adopted to identify information-rich cases with direct and sustained experience of the phenomenon under investigation. Teachers included in this study met the following criteria: (1) had taught children with IDD for at least one semester, (2) were currently teaching mathematics, and (3) conducted classroom instruction primarily through collective teaching approach. These inclusion criteria were designed to ensure that all participants shared a common experiential and pedagogical context, thereby aligning with IPA’s emphasis on exploring meaning-making within a relatively homogeneous sample.

Participants were recruited through established academic collaborations with local special schools. After obtaining institutional approval from school principals and teaching coordinators, eligible mathematics teachers were informed about the aims and procedures of the study. Teachers who met the inclusion criteria and expressed willingness to participate were then invited to join the study. Informed consent was obtained from all participating teachers, explicitly agreeing to participate in the study and to be video-recorded. Participants were clearly informed that their involvement was voluntary and that they could withdraw at any point without consequence.

Sample size determination was guided by IPA’s idiographic commitment, which prioritizes depth and richness of individual experiential accounts over breadth. A small sample was therefore deemed appropriate to allow for detailed, case-by-case analysis of teachers’ lived experiences of providing instructional support. Data collection ceased when sufficient depth and analytic richness had been achieved to meaningfully interpret participants’ experiences, consistent with established methodological guidance for IPA studies (Sparkes & Smith, 2013).

After initial consultations, five teachers volunteered to participate. The final sample size of five teachers is consistent with prior IPA research in education, where small, purposively selected samples are commonly used to facilitate in-depth phenomenological and interpretative analysis (Hennink & Kaiser, 2022). Demographic data collected from the participating teachers indicated their ages ranged from 22 to 33 years. The sample included one male teacher and four female teachers. Among these teachers, two taught first-grade classes, two taught third-grade classes, and one taught second grade. Four of the five teachers had over five years of teaching experience. Regarding educational qualifications, two teachers held master’s degrees, and three held bachelor’s degrees. Detailed demographic information of the participating teachers is summarized in Table 1.

### 2.3. Data Collection

Two complementary methods, semi-structured interviews and classroom video observations, were employed to collect comprehensive data regarding teachers’ instructional support practices for students with IDD. Consistent with the epistemological commitments of IPA, semi-structured interviews constituted the primary data source, as they provide direct access to teachers’ lived experiences and meaning-making processes. Classroom observations were used as a supplementary method to contextualize interview accounts and to inform reflective probing analytic dataset.

The classroom video observations utilized a dual-camera setup to capture a holistic view of instructional dynamics: one stationary camera positioned at the back of the classroom recorded teachers’ guidance and interactive behaviors, while a second mobile camera was strategically relocated as needed to closely document students’ behaviors and verbal interactions. Complementary audio recordings were also made to accurately capture classroom dialogs and interactions, thereby enhancing the authenticity of the recorded data. In total, 15 instructional sessions across five classrooms were documented through video recordings.

Observations were guided by an observational framework adapted from the Observation Sheet for Teacher Instructional Support in Inclusive Preschool Classroom Teaching and Learning Activities (Gong, 2022). This framework served as a heuristic tool to sensitize the researcher to salient instructional situations and interactional contexts relevant to instructional support. During a four-day preliminary phase, the observation framework was refined to better capture contextual features preceding instructional interactions, leading to the addition of a “Situation” category to document situational cues referenced later in interviews. In the formal data collection stage, video-based observations were conducted to minimize potential observer effects and to capture detailed classroom interactions (Asan & Montague, 2014). Observations focused on instructional episodes, teacher–student interactions, and classroom contexts that were subsequently used to support interpretation of interview data. Observation sessions were conducted two to three times per week, with each session lasting approximately 35 min, over a two-month period.

In addition to classroom observations, semi-structured interviews were conducted to gain deeper insights into teachers’ perceptions and interpretations of instructional support. The interview protocol was designed to elicit experiential and interpretative accounts rather than descriptive reporting of teaching practices. Drawing on prior research (Zhou et al., 2023; Hou et al., 2023), open-ended prompts encouraged participants to reflect on challenging, meaningful, or emotionally salient instructional moments. Sample prompts included questions such as: “Can you describe a moment during mathematics teaching when providing instructional support felt particularly challenging?” and “How did you experience that situation, and how do you make sense of your instructional decisions at that time?” The full interview guide is presented in Table 2. Each interview lasted approximately 40 to 50 min. Informed consent was obtained from all participants prior to the interviews. The first author led the interviews following the established guide, while the corresponding author contributed follow-up questions to explore emerging themes more deeply. With participants’ permission, interviews were audio-recorded, and field notes were taken concurrently. Reflective journal entries were completed after each interview to support reflexive engagement with the data during subsequent analysis. All interview recordings were transcribed verbatim and constituted the primary dataset for analysis. Data collection continued until sufficient depth and richness of experiential accounts had been achieved to support idiographic and interpretative analysis, rather than until thematic saturation in a statistical sense (Guest et al., 2017).

### 2.4. Data Analysis

Data analysis was conducted using IPA to explore how mathematics teachers make sense of their experiences of providing instructional support to students with IDD. In line with IPA’s idiographic and interpretative orientation, analysis proceeded through an iterative, case-by-case process prior to any cross-case comparison.

Each interview transcript was read repeatedly to achieve immersion in the participant’s account. Interview transcripts comprised over 30,000 words, while classroom video observations included 15 instructional sessions across five classrooms. All raw materials were systematically organized to support traceability throughout the analytic process and labeled using a standardized format (“Data Type + Teacher’s family name + Date”), in which interview data were marked with an “F” and video observation data with a “V” (e.g., “F-Ji-0312”; “V-Sun-0228”). Initial noting was conducted line by line, focusing on descriptive aspects of teachers’ experiences, salient language use, and preliminary conceptual interpretations. Classroom video observations were used to contextualize interview accounts and support interpretation but were not analyzed as a standalone dataset.

Emergent themes were developed within each individual case by examining connections across initial notes and identifying psychologically meaningful patterns. Each case was analyzed independently to preserve idiographic depth. Following completion of all within-case analyses, patterns of convergence and divergence were examined across cases, and related themes were clustered into higher-order superordinate themes through iterative comparison and refinement.

This analytic process resulted in five superordinate themes representing shared experiential dimensions of instructional support: (1) instructional support for comprehension, (2) instructional support for guided responses, (3) instructional support for attention, (4) instructional support for motivation, and (5) instructional support for problematic behavior management.

Throughout the analysis, interpretative movement was maintained between participants’ verbatim accounts and the researchers’ conceptual commentary, reflecting the double hermeneutic central to IPA. Reflexivity was supported through analytic memos and regular discussion among the authors to monitor assumptions and negotiate interpretations. The final analysis is presented in the Results section using illustrative interview extracts, with observational references used to enhance contextual understanding.

### 2.5. The Trustworthiness of the Findings

To ensure the trustworthiness of the study, four strategies were employed. First, credibility was enhanced through methodological triangulation by integrating video observations with interview data, enabling systematic cross-verification of emerging patterns (Heesen et al., 2019). Second, the research team engaged in collaborative analysis, holding multiple rounds of in-depth discussions to interpret codes and refine themes, thereby ensuring analytical depth and rigor. Third, reflexivity was maintained throughout the research process, with researchers regularly reflecting on their own assumptions, positionalities, and potential biases to minimize subjective interference in the analysis (Berger, 2015). Finally, member checking was conducted by returning portions of the transcribed interviews to participants for verification. This step ensured the authenticity, accuracy, and contextual sensitivity of the interpreted data (Kullman & Chudyk, 2025).

## 3. Results

From the qualitative analysis of instructional support, five themes have been identified (see Table 3). These themes stem from the goals that teachers intend to achieve through providing instructional support, corresponding to different types of such support.

### 3.1. Theme 1: Instructional Support for Comprehending Mathematical Content

#### 3.1.1. Sub-Theme 1: Explanation: Structured Explanation to Promote Conceptual Understanding

Across the five classrooms, participants experienced explanation as a deliberately regulated instructional practice aimed at making mathematical content cognitively accessible to students with IDD. Explanation was described not as simple transmission, but as an ongoing process of adjusting language and structure in response to students’ understanding.

Participants consistently emphasized the need for simplified and carefully controlled instructional language. As one teacher noted, “*Instructional language must be simple and clear*” (F-J-0312). Another teacher highlighted the tension between curricular standardization and students’ comprehension:

“*Standardized language is necessary. But while standardizing, we also need to consider students’ understanding… We need to deconstruct the standardized language or repeat it several times.*”

(F-Sun-0314)

Classroom observations showed that teachers embedded abstract mathematical ideas in familiar, everyday contexts to reduce cognitive demands. In a lesson on quantity comparison, one teacher explained:

“*The soup in this bowl only covers the bottom, while the soup in that bowl reaches the rim… this bowl has less soup, and that bowl has more.*”

(V-Chai-0302)

Such real-life analogies were experienced as essential for anchoring relational concepts such as “more” and “less” in students’ lived experiences.

Explanatory practices also varied according to students’ cognitive responsiveness. In classrooms where students demonstrated relatively stronger conceptual understanding, explanation extended to addressing misconceptions. For example, during a lesson on one-to-one correspondence, a teacher introduced a non-example:

“*Is this one-to-one correspondence here? Because the first one corresponds to two.*”

(V-Chai-0304)

Step-by-step explanation emerged as a core instructional strategy, particularly when introducing new content. Teachers described breaking tasks into sequential components to make mathematical logic explicit. In one observed lesson, a teacher modeled a comparison task:

“*First, align one end… Which pair of pants is the longest?*”

(V-Li-0305)

As one teacher explained, “*Mathematics is inherently logical… I prefer a step-by-step approach, first step one, then step two, and finally step three.*” (F-Ji-0312)

#### 3.1.2. Sub-Theme 2: Connection: Linking Mathematical Content to Real-Life and Prior Knowledge

Participants experienced connection-making as a key form of instructional support through which abstract mathematical content became meaningful for students with IDD. Rather than treating mathematics as isolated knowledge, teachers described their role as helping students situate new concepts within familiar experiences and previously learned content.

Across classrooms, teachers frequently embedded mathematical tasks in everyday contexts to reduce abstraction. For example, during a lesson on “Understanding the Number 7,” a teacher framed counting within a familiar setting:

“*There are some paintings hanging on the wall. Let’s count how many paintings there are in total.*”

(V-Li-0307)

Teachers described such contextualization to align mathematical meaning with students’ lived experiences and support engagement, particularly for students with limited abstract reasoning.

Teachers also emphasized linking new content to prior knowledge when introducing new numbers or operations. In one observed lesson, a teacher explicitly revisited earlier learning:

“*What number did we learn last time? That’s right, 1. Today, we’re learning 2.*”

(V-Shen-0228)

This approach was experienced as effective when students’ prior knowledge was stable. However, when earlier learning was fragile, teachers described the need to slow down instruction or repeatedly revisit previous concepts, revealing a tension between curricular progression and students’ actual learning pace.

#### 3.1.3. Sub-Theme 3: Questioning: Predominantly Closed-Ended Questions with Occasional Probing

Participants experienced questioning as a carefully regulated form of instructional support, primarily used to assess understanding and maintain students’ engagement during mathematics instruction. Across classrooms, closed-ended questions dominated classroom discourse, reflecting teachers’ judgment that question forms needed to align closely with students’ cognitive and linguistic capacities.

Teachers consistently expressed a preference for closed-ended formats, such as identification, binary choice, or multiple-choice questions. These formats were perceived as reducing response demands and protecting students’ sense of achievement. As one teacher explained:

“*When it comes to questioning, it shouldn’t be too difficult. We should avoid open-ended questions as much as possible… They are not conducive to students’ sense of achievement.*”

(F-Chai-0317)

Classroom observations confirmed this experience. In one lesson, a teacher asked:

“*Which pair of pants is longer, and which is shorter?*”

(V-Li-0305)

Teachers described such questions as serving a dual purpose: checking comprehension while minimizing frustration and communicative failure. Questioning, in this sense, was experienced less as a tool for exploration and more as a form of instructional scaffolding.

However, teachers’ use of questioning was not entirely uniform. When students demonstrated sufficient readiness, teachers occasionally introduced simplified probing questions to encourage reasoning and verbal expression. For example, following a successful pointing response, one teacher asked:

“*Why is this one more than that one?*”

(V-Chai-0302)

These moments reveal a tension in teachers’ experiences of questioning: while open-ended prompts were viewed as cognitively risky, they were also recognized as potentially valuable when carefully timed. Teachers thus experienced questioning as a dynamic process of balancing cognitive challenge with emotional safety, adjusting question forms in response to students’ moment-to-moment engagement and confidence.

#### 3.1.4. Sub-Theme 4: Visual Scaffolding: Supporting Conceptual Understanding Through Concrete Representations

Teachers experienced visual scaffolding as a necessary form of instructional support for helping students with IDD understand abstract mathematical concepts. Visual representations were perceived not as supplementary tools but as essential mediators between abstract ideas and students’ concrete modes of understanding.

Teachers emphasized the use of concrete objects when introducing abstract numerical concepts. As one teacher explained:

“*When dealing with a relatively abstract concept, such as the number 3, it is necessary to use three physical objects as visual cues.*”

(F-Shen-0311)

Two forms of visual scaffolding were commonly observed: visual aids and visual demonstrations. Visual aids involved presenting concrete materials directly to students, such as using pants of different lengths in a comparison task (V-Li-0305). Visual demonstrations illustrated relationships through embodied action. In one lesson on size comparison, a teacher attempted to wear a child-sized jacket and asked:

“*I am an adult. Can I wear a child’s clothes?*”

(V-Chai-0303)

Teachers described such visual strategies as effective in supporting shared understanding by making differences immediately observable. However, they also noted that the effectiveness of visual scaffolding depended on careful selection and timing, as overly complex visuals could distract students.

Overall, visual scaffolding was experienced as a context-sensitive instructional support requiring ongoing judgment to align visual representations with students’ cognitive readiness and attention.

### 3.2. Theme 2: Instructional Support for Responding the Task

#### 3.2.1. Sub-Theme 1: Visual Cues: Prompting and Scaffolding Student Responses

Participants experienced visual cues as a central form of instructional support for prompting and scaffolding students’ responses during task completion. Visual prompting was described to compensate for students’ difficulties in language production, attention, and response initiation.

Across classrooms, gestural prompts were frequently used when students hesitated or failed to respond. Teachers perceived gestures as low-demand cues that could quickly redirect attention without disrupting task flow. For example, in one observed lesson, a teacher gestured the number “1” while asking how to represent one cake, prompting a correct response (V-Sun-0311). In another instance, hand gestures were used to indicate a numerical combination when a student did not initially respond (V-Ji-0308).

When gestural cues were insufficient, teachers described escalating support through action prompts, which involved partial demonstrations without completing the task for students. As one teacher explained, “*When a student made an error in a dragging task, I traced the dragging path in the air*” (F-Ji-0312). Such prompts were intended to guide responses while preserving student agency.

In cases where students remained unresponsive, teachers resorted to full visual demonstrations. One teacher recalled, “*I asked what was different about the two bowls of rice, but the class went silent, so I raised my hand to signal them, and the students followed my gesture*” (F-Chai-0302). This progression reflects teachers’ judgment about when more explicit support was necessary to reinitiate participation.

Overall, visual cues were experienced as a flexible and adaptive form of instructional support. Teachers continuously adjusted the level of visual prompting—from subtle gestures to full demonstrations—based on students’ responsiveness, balancing accuracy, engagement, and autonomy.

#### 3.2.2. Sub-Theme 2: Language Prompts: From Minimal Cues to Modeling Support

Participants experienced language prompts as a tiered form of instructional support, adjusting verbal guidance in response to students’ responsiveness during task engagement. Teachers in this study described moving flexibly from minimal cues to more explicit verbal support when students struggled to respond.

At the initial level, teachers often repeated or rephrased questions to clarify task expectations. One teacher described how rewording a question helped reinitiate participation after initial silence:

“*I asked the question, but the whole class stayed silent. So I repeated it and rephrased it… Then one of the students responded.*”

(F-Sun-0313)

When repetition alone was insufficient, teachers introduced keyword hints or partial sentence prompts. These cues were typically brief and directive, such as “*Point to it*” when students hesitated (V-Chai-0302), or sentence stems that guided pattern-based responses, for example, completing “*Tiantian’s hat is…*” with “*smaller than Dad’s hat*” (V-Chai-0304). Teachers described these prompts as ways to reduce linguistic demands while maintaining task focus.

If students continued to struggle, teachers escalated support to modeling. This included teacher-led modeling, in which a full or simplified response was provided for students to repeat, as well as peer modeling, where a classmate’s response served as a scaffold. For instance, after a student stalled, a teacher modeled the sentence “*Snowflakes are more*,” which the student then echoed (V-Chai-0304). In classrooms with greater variability in verbal ability, modeled responses were often simplified to key terms, such as “*more*” or “*fewer*” (V-Chai-0304).

Language prompts were experienced as a graduated form of instructional support. Teachers continuously adjusted the level of verbal guidance—from minimal clarification to explicit modeling—to balance responsiveness, linguistic accessibility, and students’ active participation in mathematical tasks.

#### 3.2.3. Sub-Theme 3: Body Assistance: Embodied Support for Participation and Task Completion

Participants experienced body-based assistance as a necessary form of instructional support when verbal and visual prompts were insufficient to elicit student participation or task completion. Bodily involvement was described to translate abstract instruction into concrete, felt experiences, particularly for students with limited verbal comprehension.

One form of support involved experiential body prompts, in which teachers physically enacted concepts to help students understand required responses. As one teacher explained:

“*One time, a student gave an irrelevant answer, so I picked up a hat and placed it on his head… to help him understand through the experience.*”

(F-Chai-0303)

Teachers described such embodied prompts as enabling students to grasp meaning through direct physical experience.

A more intensive form of support involved hand-over-hand assistance, typically used when students were unable to complete tasks independently. For example, one teacher noted:

“*After showing the steps a few times, I ended up guiding the student’s hand myself—helping them drag the pencil across the screen and pick the right options.*”

(F-Li-0305)

Similar assistance was observed when a teacher guided a student hand-by-hand during a counting task (V-Sun-0311). Teachers described this form of support as necessary for sustaining participation, while also recognizing it as a temporary measure when independent responding was not yet possible.

### 3.3. Theme 3: Instructional Support for Maintaining Attention

#### 3.3.1. Sub-Theme 1: Individual Support: Prevention Combined with Reminders

Participants experienced individual support for maintaining attention as a combination of preventive strategies and timely reminders, used to sustain students’ engagement during mathematics instruction. Preventive support was often applied before distraction fully emerged, reflecting teachers’ anticipation of attention difficulties.

A commonly described preventive strategy involved calling students by name or using brief auditory cues to recapture focus. As one teacher explained:

“*When their collective focus wanes, I call out a student’s name or tap the desk or blackboard to create a sound that recaptures attention.*”

(F-Ji-0312)

Classroom observations confirmed this practice, such as when a teacher redirected a distracted student by saying, “*X, look here, sit properly, face the front*” (V-Li-0305).

Teachers also used physical proximity as a preventive form of attention support. By moving closer to students, modeling responses, or demonstrating key concepts, teachers sought to maintain engagement and reduce off-task behavior. For example, during a reading activity, a teacher approached each student’s seat and modeled the target phrase when it was their turn (V-Li-0307). Similar practices were observed during group work, where teachers walked to students’ desks and demonstrated concepts such as “*more*” and “*less*” (V-Sun-0301).

When attention had already shifted, teachers applied responsive reminders to quickly re-engage students. One teacher described using brief verbal prompts to restore focus:

“*I noticed one of the students zoning out, so I quickly asked, ‘Doing addition, think?’—and that brought him right back.*”

(F-Shen-0312)

From participants’ perspectives, individual attention support was experienced as a moment-to-moment adjustment process. Teachers balanced anticipation and response, using brief, targeted interventions to maintain attention while minimizing disruption to ongoing instruction.

#### 3.3.2. Sub-Theme 2: Collective Attention: Routine-Based Support

Participants experienced collective attention support as relying primarily on routine-based practices that helped establish and sustain shared focus during mathematics lessons. Rather than responding to individual distraction alone, routines were described as providing a predictable attentional framework for the whole class.

Engaging lesson openings were commonly used to orient collective attention at the start of instruction. For example, one teacher introduced the concepts of “bigger” and “smaller” through an animation comparing adults’ and children’s objects (V-Chai-0303), while another used a short narrative about carrots and bread to initiate a lesson on the number 9 (V-Ji-0310).

Choral chants and classroom rituals were also widely used to regulate transitions and maintain joint attention. As one teacher explained:

“*We use collective commands like ‘1, 2, 3, do well… little eyes on the teacher’; or when counting, ‘little hands ready, prepare, 1, 2, 3.’*”

(F-Ji-0312)

Teachers experienced these routine-based practices as efficient collective support that reduced disruption and the need for repeated individual reminders, embedding attentional regulation into predictable classroom activities.

#### 3.3.3. Sub-Theme 3: Content-Based Focus: Multi-Modal and Layered Cues

Participants experienced content-based focus as relying on layered audio-visual cues to direct students’ attention to key instructional information. These strategies included tapping the blackboard, sequential presentation of content, pointing with teaching aids, and modulating vocal delivery. All five participants employed at least one such strategy.

Classroom observations showed that teachers often combined visual and auditory cues to structure attention. In one lesson, “*while explaining a sentence on the multimedia screen, the teacher used a pointer to direct student gaze to specific visual elements*” (V-Chai-0302). In another, “*a teacher tapped the board before pointing to equations for students to read in unison*” (V-Li-0309). Sequential presentation was also used to scaffold attention, such as when reviewing number bonds of 9 step by step (V-Ji-0310).

When students showed limited auditory responsiveness, teachers adjusted vocal tone and pacing to emphasize key content. For example, one teacher slowed her speech and stressed keywords such as “more” and “less” using exaggerated intonation (V-Chai-0304).

Content-based focus was experienced as a targeted instructional support in which teachers layered visual and auditory cues to stabilize attention on key mathematical ideas.

### 3.4. Theme 4: Instructional Support for Sustaining Motivation

#### 3.4.1. Sub-Theme 1: Encourage and Praise

Participants experienced encouragement and praise as a primary form of instructional support for sustaining students’ motivation during mathematics learning. This support was most often delivered through immediate verbal affirmations. As one teacher noted, “*When a student got it right, I praised her and said, ‘Very good! She answered wonderfully!*’” (F-Chai-0302). Verbal praise was frequently combined with symbolic gestures, such as a thumbs-up, to enhance its impact (V-Ji-0308).

Beyond individual praise, teachers also employed collective forms of recognition to foster a positive classroom climate. For example, after a student finished reading, “the teacher said, *‘M, your voice was so clear! Let’s all praise him!’, followed by the entire class clapping*” (V-Ji-0308). Teachers likewise encouraged self-praise to build shared morale, as observed when a teacher said, “*Let’s give ourselves a round of applause for every one of you*” (V-Ji-0308).

While immediate praise was most common, delayed rewards were occasionally used. For instance, “*after completing seatwork exercises, students were rewarded with a multimedia game activity*” (V-Ji-0308). Teachers viewed such delayed incentives as supplementary support for sustaining motivation during longer tasks.

Encouragement and praise were experienced as flexible motivational support. Teachers adjusted reinforcement strategies—individual, collective, or delayed—according to students’ responsiveness and classroom dynamics.

#### 3.4.2. Sub-Theme 2: Try Again

Participants experienced “try again” as an important form of motivational support when students responded incorrectly. Teachers described intentionally providing students with a second opportunity to engage with the task in a supportive manner. As one teacher explained:

“*If the answer is wrong, I might say, ‘You’ve tried really hard, but think about it again. How should we solve this problem?’ instead of saying, ‘This is wrong.’ Otherwise, the latter would reinforce a negative response.*”

(F-Sun-0311)

Teachers perceived this approach as protecting students’ emotional safety while encouraging continued participation. Classroom observations illustrated how second-chance opportunities were paired with gentle instructional guidance. For example, after an incorrect response, a teacher redirected the student by saying:

“*Stand a little further back and see which bowl of soup is fuller.*”

(V-Chai-0302)

### 3.5. Theme 5: Instructional Support for Regulating Behavior

#### 3.5.1. Sub-Theme 1: Verbal Restraint

Participants experienced verbal restraint as a primary form of instructional support for regulating emerging behavioral issues during mathematics instruction. Verbal prompts were used to address both individual behaviors and collective classroom order.

When behavioral issues involved individual students, teachers commonly issued brief, direct verbal warnings. As one teacher explained, “*when a student made disruptive noises, I would directly address them by name, ‘M, be quiet’. The student then stopped the behavior*” (F-Chai-0302). Teachers viewed such individualized restraint as an efficient way to interrupt disruptive behavior without escalating tension.

When multiple students exhibited off-task behavior, teachers reiterated classroom rules to restore collective order. Classroom expectations were explicitly restated, as observed in one lesson:

“*In math class, first, no lying on the desks; second, no moving books around; third, answer questions with a loud and clear voice.*”

(V-Ji-0308)

Teachers also described allowing brief breaks when students were unable to self-regulate due to emotional distress. One teacher explained how she addressed the class while supporting the distressed student:

“*M is feeling a bit unwell and needs a moment to calm down, so let him take a break. But what should the rest of you do? Listen carefully to the teacher.*”

(F-Sun-0312)

Taken together, verbal restraint was experienced as a flexible regulatory support. Teachers adjusted their verbal responses—from direct warnings to rule reminders and temporary breaks—according to students’ emotional states and classroom context, aiming to maintain order while minimizing disruption.

#### 3.5.2. Sub-Theme 2: Physical Interventions

When verbal strategies were insufficient, teachers selectively employed physical interventions to regulate student behavior. These interventions were described as escalating support, used cautiously to restore order while minimizing disruption.

At a minimal level, teachers used physical gestures to signal behavioral expectations. For example, “*When a student spoke out without permission, I simply gave a quiet hand gesture to signal silence, and the student stopped right away*” (F-Ji-0308). Proximity control was also commonly used, with teachers approaching disengaged students to prompt adjustment. As observed, “*A student slouched on their desk, and the teacher approached the student to remind him, leading the student to adjust his posture*” (V-Ji-0308).

When more direct action was required, teachers employed gentle tactile cues. One teacher explained, “*I tried reminding him verbally, but he didn’t respond. So I walked over and gently guided him back to his seat*” (F-Shen-0308). In urgent situations involving potential harm, teachers intervened physically to prevent escalation, such as taking a student’s arm and stating, “*Keep your hands to yourself*” (V-Sun-0312).

Temporary isolation was used as a last resort. For instance, “*a student began crying uncontrollably and was escorted by an assistant to a quiet area at the back of the classroom to regain composure*” (V-Ji-0308).

## 4. Discussion

This study examined the instructional support as experienced and enacted by mathematics teachers in Chinese special schools serving students with IDD. The findings revealed a five-domain support framework: comprehending mathematical content, responding to tasks, maintaining attention, sustaining motivation, and regulating behavior. From an interpretative phenomenological perspective, these domains represent how teachers make sense of instructional support in response to students’ cognitive and behavioral challenges. Specifically, the framework reflects teachers’ experiential understanding of what it means to “teach mathematics effectively” in classrooms characterized by limited abstract reasoning, emotional vulnerability, and fluctuating engagement. In this sense, the framework directly addresses Research Question 1 and Research Question 2 by linking teachers’ lived experiences to their instructional interpretations. The framework shows conceptual overlap with Vogt et al.’s (2021) individual support model and Pianta et al.’s (2012) instructional quality framework, particularly in relation to explanation, feedback-related support, and motivation. This overlap suggests that teachers’ meaning-making remains anchored in core principles of effective instruction. At the same time, the prominence of attention regulation and behavior management reflects teachers’ lived experience of instruction in Chinese special schools. In these contexts, maintaining engagement and behavioral stability is perceived as inseparable from teaching itself. This finding highlights the contextual and cultural specificity of instructional support and underscores the contribution of this IPA study in illuminating how teachers interpret and navigate instructional demands within specialized educational settings.

This study found that, in supporting students’ comprehension, special education teachers exhibited both similarities and differences compared to their general education counterparts. Shared strategies such as explanation, making content connections, and questioning were frequently used to scaffold students’ understanding. However, teachers in this study primarily interpreted these strategies as means of ensuring accessibility and task completion, rather than as tools for fostering autonomy or creativity. This divergence is primarily rooted in the cognitive characteristics of students with IDD, whose learning capacities and developmental trajectories often differ significantly from those of their neurotypical peers (Hassiotis, 2000). For example, questioning practices were predominantly teacher-led and focused on factual or closed-ended questions. Teachers experienced this format as necessary for maintaining engagement and preventing frustration, a pattern that aligns with Zhu et al.’s (2023) findings on instructional discourse in Chinese special schools. Although occasional efforts were made to elicit individual student opinions, these were limited in scope and revealed a generally reduced focus on learner agency. This tendency reflects the broader developmental profile of students with IDD, many of whom remain at the concrete and representational stages of cognitive functioning and exhibit substantial difficulties in abstract reasoning, particularly in internalizing mathematical symbols and understanding logical relationships. Consequently, students relied heavily on tangible and visual supports, including manipulatives, diagrams, and physical demonstrations, to construct meaning. The frequent use of such strategies reinforces the relevance of multisensory and stage-based approaches, such as the Concrete–Representational–Abstract (CRA) model. Prior research has been shown that CRA effectively promote conceptual understanding among students with disabilities (E. Bouck et al., 2017; Ruştioğlu & Avcıoğlu, 2022). The consistent application of hands-on materials and visual cues observed in these classrooms provides further empirical support for the applicability of CRA-informed practices in special education mathematics instruction.

The findings indicated that instructional support for responding to the task in special education classrooms followed a progressive structure, ranging from minimal prompts to full demonstrations. Teachers experienced this progression as a practical way of calibrating support in response to students’ moment-to-moment engagement and performance, rather than as a fixed instructional sequence. This scaffolding approach aligns with Vygotsky’s concept of the Zone of Proximal Development (ZPD), emphasizing adaptive support based on student responsiveness (Eun, 2019). The study also revealed differentiated support across student ability tiers. For students in tiers A and B (better ability), teachers maintained higher expectations and provided limited prompts to encourage autonomy, whereas students in tier C (lower ability) received more direct guidance, including hand-over-hand assistance. From an experiential perspective, teachers interpreted such differentiation as a necessary response to students’ perceived learning readiness and emotional tolerance for challenge. However, this pattern also raises concerns consistent with the “Matthew Effect”. Higher-achieving students may receive more sustained support, while lower-performing peers risk instructional neglect (Larsen & Little, 2023). This finding also has been documented in Wan’s (2023) research, which found that teachers sometimes reduced or withdrew support prematurely when students failed to respond. Taken together, these findings highlight a tension experienced by teachers between fostering autonomy and ensuring access. They underscore the importance of sustained and equitable scaffolding for students with greater learning needs throughout the instructional process.

Teachers in special education settings experienced encouragement and praise as core strategies for sustaining motivation. These practices were often supplemented by gamified incentives and symbolic rewards, consistent with prior empirical evidence (Lin, 2022). Verbal praise, particularly non-specific and repetitive forms such as “Great, great,” was frequently observed. This echoes findings from discourse analyses of special education classrooms (X. Huang, 2023). Teachers did not consistently differentiate between praise types. Instead, they described praise primarily as a means of maintaining emotional safety and participation. Importantly, teachers typically avoid direct criticism when students provide incorrect answers. Participants interpreted this restraint as a protective strategy, reflecting an awareness of neurodiversity and students’ heightened sensitivity to evaluative feedback, which can undermine self-regulation and persistence (Begley & White, 2003). From an IPA perspective, these practices illustrate how teachers understand motivation not as an internal trait to be cultivated, but as a fragile state that must be carefully sustained through emotionally supportive instructional interactions. Rather than emphasizing correction, teachers offer additional response opportunities paired with guided support. They experienced this approach to preserve motivation while allowing students to re-engage with mathematical content without experiencing failure.

In terms of maintaining attention, this study found that special education teachers adopted a dual-pronged approach to support students with IDD. Proactive strategies, such as animated stimuli, blackboard highlighting, and visual cues, were used to sustain engagement, while reactive verbal redirection was applied when students became distracted. Teachers experienced this combination of proactive and reactive strategies as necessary for managing students’ fluctuating attentional states within highly structured classroom environments. These strategies reflect a flexible and responsive teaching style that adapts to students’ attentional variability. They are consistent with Lin’s (2022) finding on the use of visual and verbal prompts in special classrooms. Moreover, the frequent use of collective verbal routines further supported group-based attention and reinforced behavioral norms. Participants interpreted these routines as providing predictability and shared structure, helping students remain oriented to classroom expectations. These collective routines reflect broader sociocultural expectations within Chinese classrooms, where synchronized participation and collective discipline are experienced by teachers as essential conditions for instructional support. Rather than framing these practices in opposition to Western approaches, the findings illustrate how attention regulation is culturally embedded and experienced by teachers as inseparable from instructional support in Chinese special school contexts.

Finally, this study found that strategies for regulating student behavior were frequently integrated into instructional support for students with IDD. These strategies were served to restore learning conditions and sustain classroom engagement. Teachers adopted tiered responses based on behavioral severity, ranging from verbal warnings and physical proximity for minor disruptions to time-out and removal to calming areas for more intense episodes. These findings are consistent with previous studies indicating that students in special classes often exhibit higher rates of behavioral challenges that disrupt instructional flow. While some scholars have categorized such control-oriented practices as non-instructional (Q. Huang, 2020), the current study highlights their pedagogical function in special education settings. These strategies not only ensure short-term classroom order but also facilitate students’ re-engagement with academic tasks, particularly in emotionally charged situations.

## 5. Conclusions

This study delineates the instructional support and implementation processes employed by mathematics teachers in special schools. Grounded in teachers’ experiential efforts to address both cognitive access and classroom engagement, teachers demonstrated five principal support modalities: instructional support for comprehending mathematical content, instructional support for responding to tasks, instructional support for maintaining attention, instructional support for sustaining motivation and instructional support for regulating behavior. Together, these five modalities directly address Research Question 1 by identifying the forms of instructional support enacted in Chinese special school mathematics classrooms, and Research Question 2 by illustrating how teachers adapt these supports in response to students’ cognitive and behavioral challenges. Overall, teachers exhibited adaptive responsiveness in tailoring supports to students’ cognitive capacities and classroom performance. At the same time, the findings reveal tensions within teachers’ instructional decision-making, particularly in balancing accessibility, emotional regulation, and opportunities for autonomy, highlighting areas for reflective professional development in practice.

These findings have important implications for teacher training and curriculum design in special education. Professional development programs may benefit from supporting teachers in expanding repertoires of questioning, scaffolding, and motivational strategies while remaining responsive to students’ developmental profiles. Similarly, curriculum design in special schools could integrate flexible instructional frameworks that recognize attention regulation and behavior support as integral components of mathematics instruction.

This study has several limitations. First, although a limited number of lessons were observed for each teacher, which constrained the range of instructional situations captured, the design enabled in-depth engagement with teachers’ lived experiences of instructional support. Variations in lesson types and content across classrooms may therefore influence the interpretation of findings. Future research could focus on life mathematics classrooms with comparable lesson content within the same grade level to enhance analytic consistency. Second, while this study examined instructional support at the whole-class level, the marked heterogeneity among students in special education classrooms suggests that teachers may adjust strategies according to students’ specific disabilities. The present analysis prioritized teachers’ experiential accounts rather than systematic comparisons across disability categories. Future studies could disaggregate student groups to generate more targeted pedagogical insights. Finally, as a qualitative study grounded in IPA, the findings are inherently context-bound and not intended for statistical generalization. Instead, they offer interpretative and theoretical insights into how instructional support is understood and enacted within Chinese special education settings. By applying IPA in this context, the study contributes to emerging phenomenological research on culturally situated educational practice in China (Cutri, 2025) and provides a foundation for future comparative and longitudinal inquiry.

## Figures and Tables

**Table 1:** The demographics of participants (n = 5).

Participant	Age	Gender	Class	Degree	Years of Teaching Experience
SUN	32	Male	Grade 1	Bachelor	10
CHAI	33	Female	Grade 1	Master	7
LI	22	Female	Grade 2	Bachelor	1
JI	34	Female	Grade 3	Master	8
SHEN	29	Female	Grade 3	Bachelor	7

**Table 2:** Semi-structured interview questions.

No.	Questions
1	In the teaching of Life Mathematics for students with intellectual disabilities, what aspects of instructional support do you think should be provided to the students?
2	Under what specific circumstances do you usually provide instructional support to the students?
3	What kind of instructional support do you usually provide to the students?
4	How do you provide instructional support to the students?
5	When you provide instructional support to the students in the teaching of Life Mathematics, what difficulties or obstacles have you encountered?
6	In the teaching of Mathematics for students with intellectual disabilities, what factors do you think will affect teachers’ instructional support behaviors towards students?

**Table 3:** The themes and subthemes in the current study.

Theme	Subthemes
Theme 1: Instructional support for comprehending mathematical content	1.1 Explanation: Structured explanation to promote conceptual understanding
	1.2 Connection: Linking mathematical content to real-life and prior knowledge
	1.3 Questioning: Predominantly closed-ended questions with occasional probing
	1.4 Visual scaffolding: Supporting conceptual understanding through concrete representations
Theme 2: Instructional support for responding to the task	2.1 Visual cues: prompting and scaffolding student responses
	2.2 Language prompts: from minimal cues to modeling support
	2.3 Body assistance: embodied support for participation and task completion
Theme 3: Instructional support for maintaining attention	3.1 Individual Support: prevention combined with reminders
	3.2 Collective attention: routine-based support
	3.3 Content-based focus: multi-modal and layered cues
Theme 4: Instructional support for sustaining motivation	4.1 Encourage and praise
	4.2 Try again
Theme 5: Instructional support for regulating behavior	5.1 Verbal restraint
	5.2 Physical interventions

## Data Availability

The data presented in this study are available on request from the corresponding author due to our promise in the informed consent form that the data would be kept confidential.
